# Hypoxia-Inducible Factor 1-Alpha (HIF-1α) and Cancer: Mechanisms of Tumor Hypoxia and Therapeutic Targeting

**DOI:** 10.7759/cureus.70700

**Published:** 2024-10-02

**Authors:** Mohd Basheeruddin, Sana Qausain

**Affiliations:** 1 Biochemistry, Jawaharlal Nehru Medical College, Datta Meghe Institute of Higher Education and Research, Wardha, IND

**Keywords:** angiogenesis, cancer progression, hif-1α, immunotherapy, rna interference, small molecule inhibitors, therapeutic targeting

## Abstract

Hypoxia-inducible factor 1-alpha (HIF-1α) is necessary for cells to adapt to low oxygen levels often present in the tumor microenvironment. HIF-1α triggers a transcriptional program that promotes invasion, angiogenesis, metabolic reprogramming, and cell survival when it is active in hypoxic environments. These processes together lead to the growth and spread of tumors. This review article examines the molecular mechanisms by which HIF-1α contributes to tumor progression, including its regulation by oxygen-dependent and independent pathways, interactions with oncogenic signaling networks, and impact on the tumor microenvironment. Additionally, we explore current therapeutic strategies targeting HIF-1α, such as small molecule inhibitors, RNA interference, and immunotherapy approaches. Understanding the multifaceted roles of HIF-1α in cancer biology not only elucidates the complexities of tumor hypoxia but also opens avenues for developing novel and more effective cancer therapies.

## Introduction and background

Overview of hypoxia-inducible factor 1-alpha and cancer

**Figure 1:**
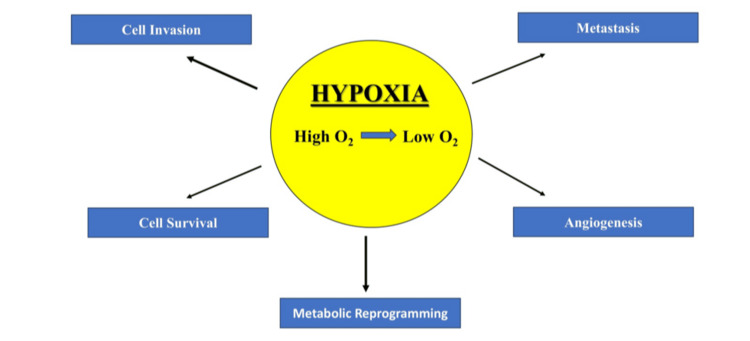
Schematic representation of hypoxia-inducible factor 1-alpha (HIF-1α) activation under hypoxic conditions, illustrating its role in promoting tumor development. Image credit: Sana Qausain.

Hypoxia, or the lack of oxygen in tissues, is a hallmark of the tumor microenvironment that has a large impact on treatment resistance and the advancement of cancer. Hypoxia-inducible factor 1-alpha (HIF-1α) is a transcription factor at the forefront of the cellular response to hypoxia. Cancer cells need to be able to adapt and survive in low-oxygen environments [1]. Figure 1 shows how HIF-1α activation promotes tumor development and aggressiveness by triggering the transcription of many genes that aid in angiogenesis, metabolic reprogramming, cell survival, invasion, and metastasis.

In cancer, HIF-1α is often upregulated not only due to hypoxic conditions but also through genetic alterations that stabilize and activate the protein regardless of oxygen levels. Mutations in oncogenes and tumor suppressor genes can lead to enhanced HIF-1α activity, driving a more malignant phenotype and contributing to poor clinical outcomes. The pervasive influence of HIF-1α in cancer underscores its importance as a key regulator of the hypoxic response and as a critical player in tumor biology [2]. The central role of HIF-1α in promoting tumor progression makes it an attractive target for cancer therapy. Therapeutic strategies aimed at inhibiting HIF-1α include small molecule inhibitors, RNA interference, and novel immunotherapeutic approaches. These interventions seek to disrupt the pathways that HIF-1α regulates, thereby impairing the tumor’s ability to adapt to hypoxia, reducing angiogenesis, and sensitizing cancer cells to conventional treatments [3].

Goal of the review

This review article aims to present a thorough summary of the processes via which HIF-1α promotes tumor hypoxia and growth. The available treatment approaches that target HIF-1α will be covered. By elucidating the complex interplay between HIF-1α and cancer, this review seeks to highlight potential avenues for novel and more effective cancer therapies, ultimately contributing to improved patient outcomes.

## Review

Mechanisms of tumor hypoxia

When the oxygen supply from existing blood vessels has not kept up with the rapid growth of the tumor, an imbalance between oxygen use and supply occurs, leading to tumor hypoxia. In low-oxygen environments, HIF-1α stabilizes, eluding degradation and migrating to the nucleus, where it dimerizes with HIF-1β. This complex attaches to target gene promoter regions’ hypoxia-responsive elements (HREs) to start the transcription of several genes that support hypoxia adaption [4].

Rapid Tumor Growth and Insufficient Angiogenesis

Rapid growth frequently surpasses the blood supply of tumors. Tumors drive angiogenesis, the process by which pre-existing blood vessels divide to generate new ones, although the resulting vasculature is usually abnormal and dysfunctional [5]. Tumor blood vessels are frequently leaky, twisted, and disorganized, which results in an ineffective oxygen supply. As a result, some tumor areas experience hypoxia or low oxygenation. Although it encourages angiogenesis, vascular endothelial growth factor (VEGF) can also result in aberrant vasculature. Additionally, insufficient perfusion and blood flow irregularities contribute to the development of hypoxic regions [6].

Increased Oxygen Consumption

Cancer cells exhibit increased metabolic activity and oxygen consumption to support their rapid proliferation. This heightened oxygen demand further exacerbates the oxygen deficit in the tumor microenvironment. This elevated metabolic requirement is further influenced by the Warburg effect, a process in which cancer cells preferentially use glycolysis for energy synthesis even in the presence of oxygen. VEGF promotes angiogenesis but results in abnormal vasculature. Insufficient perfusion and blood flow irregularities contribute to hypoxic regions [7].

Structural Abnormalities in Tumor Vasculature

The structural abnormalities of tumor vasculature not only lead to poor oxygen delivery but also create heterogeneous oxygen distribution within the tumor. Some regions receive adequate oxygen supply, while others remain hypoxic. The chaotic structure of the tumor blood vessels contributes to this uneven oxygen distribution. Irregular and inefficient blood vessel formation leads to heterogeneous oxygen distribution due to vessel dysfunction [8].

High Interstitial Fluid Pressure

Tumors often have elevated interstitial fluid pressure due to the leaky nature of their blood vessels and the lack of effective lymphatic drainage. This high interstitial fluid pressure impedes the flow of oxygenated blood into the tumor mass, further contributing to hypoxia. Leaky blood vessels increase interstitial fluid pressure. Impaired lymphatic drainage exacerbates fluid accumulation and pressure [9].

Cellular Adaptations to Hypoxia

Cancer cells activate HIF-1α in response to hypoxia, which sets off a transcriptional cycle that facilitates survival and adaption to low oxygen situations. Cancer cells can proliferate even in hypoxic environments due to the activation of genes related to angiogenesis, metabolic reprogramming, cell survival, and invasion. Low oxygen levels are stabilized by HIF-1α activation. As a result, genes that are adaptive to deal with hypoxia are upregulated [10].

Hypoxia-induced angiogenesis

**Figure 2:**
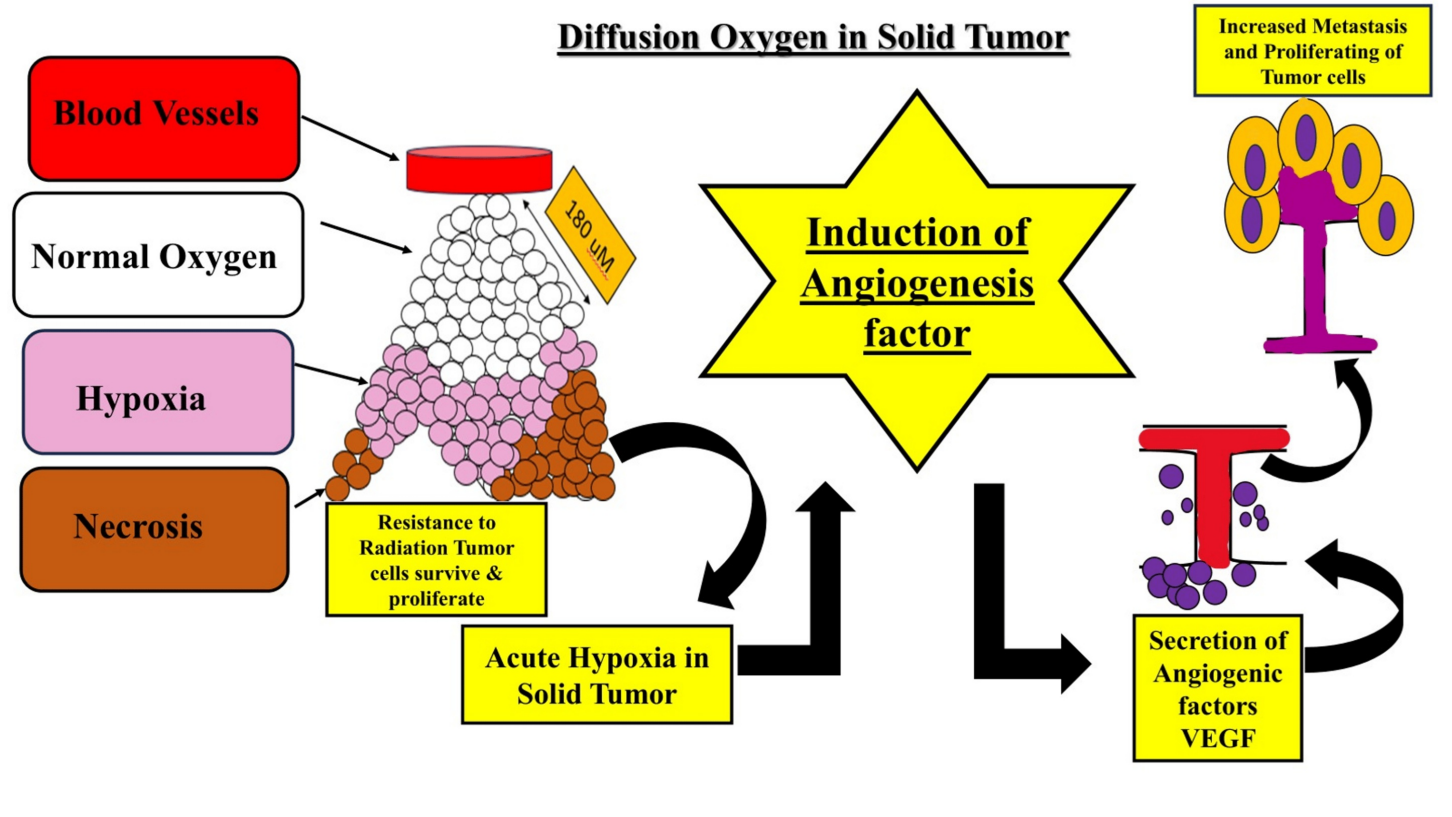
Hypoxia-induced angiogenesis driven by hypoxia-inducible factor 1-alpha (HIF-1α) and multiple signaling pathways. Image credit: Sana Qausain.

Figure 2 illustrates hypoxia, a state of low oxygen levels that is frequently present in the tumor microenvironment. A crucial transcription factor known as HIF-1α is involved in cellular responses to hypoxia, including the stimulation of angiogenesis, the process by which new blood vessels grow out of pre-existing ones. Because it provides the quickly reproducing cancer cells with the oxygen and nutrients they need, angiogenesis is essential for the growth and survival of tumors. Hypoxia-induced angiogenesis involves a complex interplay of signaling pathways and molecular mechanisms driven by HIF-1α and other factors [11].

Activation of Hypoxia-Inducible Factor 1-Alpha Under Hypoxic Conditions

Prolyl hydroxylase domain proteins (PHDs) hydroxylate HIF-1α under normoxic circumstances, which then triggers the von Hippel-Lindau (VHL) protein to recognize it and then ubiquitinate and protease it. Hypoxic conditions, on the other hand, cause PHDs to become inactive, which stabilizes and accumulates HIF-1α. Once stabilized, HIF-1α moves into the nucleus, where it dimerizes with HIF-1β and attaches itself to HREs found in the target gene promoter regions to start the transcription of those genes [12].

Upregulation of Pro-angiogenic Factors

The overexpression of several pro-angiogenic factors is one of the main consequences of HIF-1α activation. VEGF, a powerful inducer of angiogenesis, is the most well-known of these. VEGF and other angiogenic factors, including fibroblast growth factors (FGFs), angiopoietins, and platelet-derived growth factor (PDGF), are all induced to be expressed by HIF-1α [13].

Vascular endothelial growth factor: VEGF is a protein that can actively contribute to forming new blood vessels by stimulating endothelial cell division and movement to the zones that demand angiogenesis. It also helps maintain endothelial cell viability under any circumstance. It is integral for tissue remodeling and wound repair [14].

Platelet-derived growth factor: PDGF is essential in producing and organizing new blood vessels by attracting smooth muscle cells and pericytes that help stabilize them. It is fundamental in determining the pattern and mechanical properties of neovasculature. PDGF is also known to improve the well-being and function of the blood vessels [15].

Angiopoietins: These are crucial proteins in making and stabilizing new and existing blood vessels, a process called angiogenesis. They assist in calling other supporting cells to repair and stabilize vessel walls. Angiopoietins play a crucial role in the protection and maintenance of the stability and functionality of the vascular network. The Inko8 protein group plays an essential role in forming, repairing, and maintaining vasculature [16].

Fibroblast growth factors: FGFs are indispensable proteins that stimulate the growth and division of endothelial cells fundamental to angiogenesis and blood vessel development. They also participate in the restoration of tissues by maintaining the strategic vascular plexus density. FGFs are now known to play an important role in many aspects of wound healing and management of tissues. They are generally involved in important processes such as angiogenesis, supporting blood circulation, and tissue remodeling to maintain body health [17].

Endothelial Cell Activation and Migration

By binding to their respective receptors on endothelial cells, the pro-angiogenic molecules increased by HIF-1α set off a series of signaling cascades that facilitate endothelial cell activation, proliferation, migration, and survival. For example, VEGF interacts with its receptors on endothelial cells (VEGFR-1 and VEGFR-2), triggering downstream signaling pathways such as the mitogen-activated protein kinase (MAPK) and phosphatidylinositol 3-kinase/Akt pathway. These pathways facilitate endothelial cell migration toward the hypoxic regions of the tumor, where new blood vessels are needed [18].

PI3K/Akt pathway: One of the most significant signaling pathways involved in cell survival signal and promoting cell migration is PI3K/Akt. This protein has a significant function in disrupting the process of intended cell death and bookkeeping cellular replies to pressure. This pathway also helps in the transport of cells to areas within the body where they are needed. It is required for normal cellular growth and repair [19].

MAPK pathway: One of the essential routes that control cell growth and migration is the MAPK pathway. It aids in the cell growth control and the distribution of cells in certain regions. This pathway is very important in interpreting received signals from the surroundings and in the development of tissues. It has to be activated for its functions in multiple cellular processes, such as repair and adaptability [20].

Extracellular Matrix Remodeling

The extracellular matrix (ECM) must be remodeled for endothelial cells to migrate and invade the surrounding tissue. HIF-1α upregulates the expression of matrix metalloproteinases (MMPs), which degrade components of the ECM, allowing endothelial cells to invade and form new blood vessels. Additionally, lysyl oxidase (LOX), another target of HIF-1α, cross-links collagen and elastin in the ECM, providing structural support for the newly formed vasculature [21].

Matrix metalloproteinases: MMPs are metalloenzymes capable of degrading parts of ECM to facilitate endothelial cell invasion to other tissues. This is important in the process of angiogenesis and remodeling of tissues. MMPs, therefore, degrade ECM to allow cells to move and get to the desired structures. Its activity is required for various physiological processes, including those that are part of healing impaired tissues, such as wounds, or the formation of new blood vessels, angiogenesis [22].

Lysyl oxidase: LOX is an enzyme that stabilizes newly formed blood vessels by covalently modifying the ECM scaffold. This cross-linking gives mechanical properties and strength to the vessels that are formed and further developing vasculature. LOX activity is required for the stabilization and proper functioning of newly formed vessels. It is active in such steps as the ones of tissue remodeling and vessel integration [23].

Formation of New Blood Vessels

The coordinated actions of endothelial cell proliferation, migration, and ECM remodeling culminate in the formation of new blood vessels, a process known as neovascularization. These newly formed vessels initially exhibit a disorganized and immature structure, often leading to inefficient blood flow and further regions of hypoxia. Over time, additional signaling molecules such as angiopoietins and PDGF contribute to the maturation and stabilization of the new vasculature [24].

Neovascularization: Neovascularization is the process of new vessel formation from pre-existing vessels. This is important in meeting the need for oxygen as well as nutrients to tissues, for example, during growth, healing, or development. One essential form of angiogenesis is characterized by an increase in endothelial cell numbers and their mobility that results in the development of new vessels. Angiogenesis is critical in wound healing and, in general, in reaction to injury or ischemia [5].

Vessel maturation: MVD leads to the maturation of new blood vessels by augmenting further signaling by other molecules within the blood vessel. This process ensures that the vessels get to develop their structural complement and functionality. Chemical messengers map the place and time of the arrival of other auxiliary cells as well as control vessel formation and maturation. These changes exert profound influence over numerous tissues and organ systems in outcomes pertinent to vascular health; further, maturation is essential for sustaining the integrity of the endothelial layers [25].

Metabolic reprogramming in cancer

Metabolic reprogramming is a hallmark of cancer that enables tumor cells to adapt to the challenging microenvironment, including low oxygen levels (hypoxia) and nutrient scarcity. This adaptive response is driven by various factors, with HIF-1α playing a central role in orchestrating these metabolic changes. Understanding metabolic reprogramming is crucial for developing targeted cancer therapies [26].

The Warburg Effect

**Figure 3:**
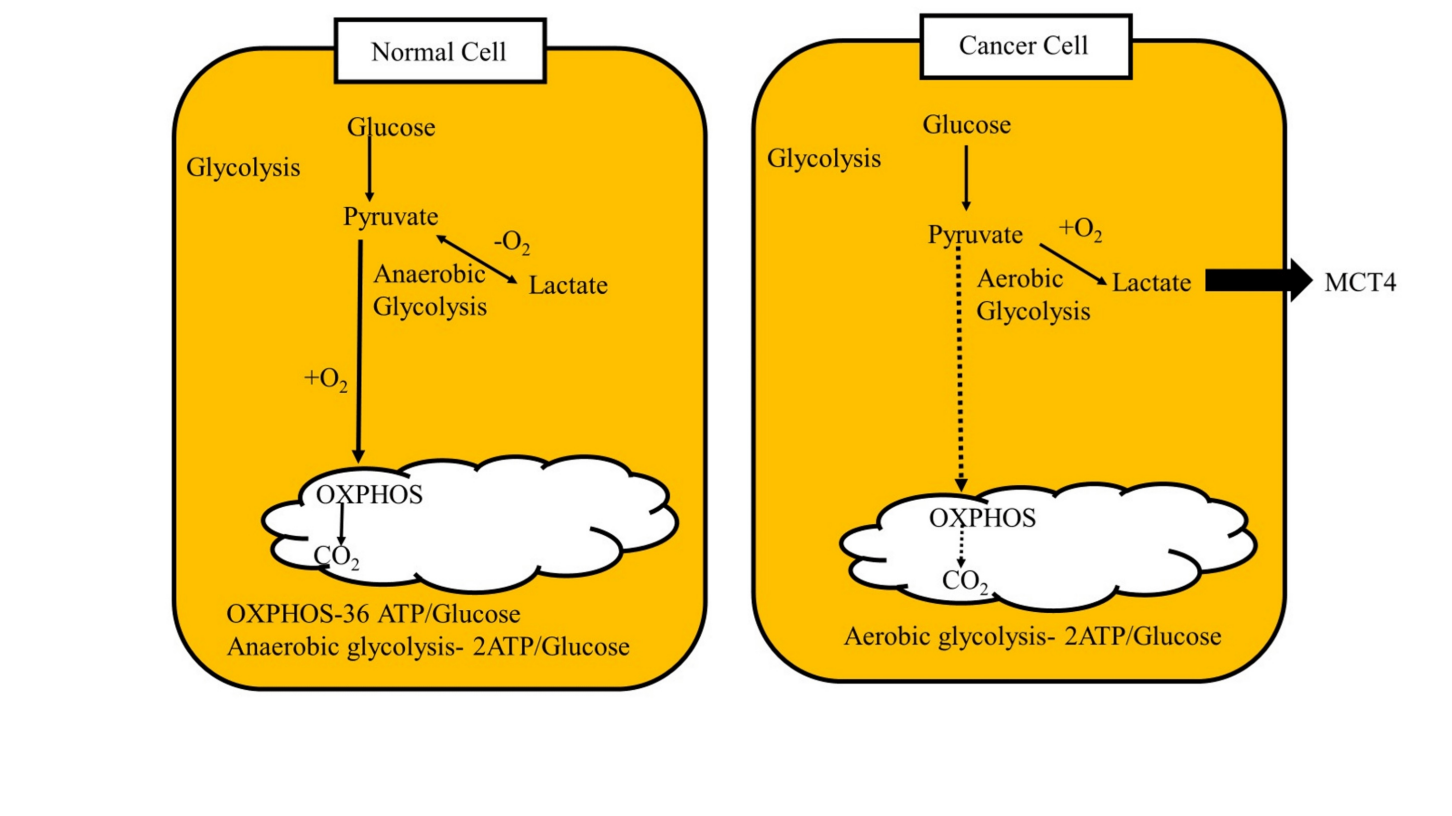
Metabolic reprogramming in cancer cells via the Warburg effect, emphasizing enhanced glycolysis. OXPHOS: oxidative phosphorylation Image credit: Sana Qausain

One of the most well-known features of cancer metabolism is the Warburg effect (Figure 3), characterized by a preference for glycolysis over oxidative phosphorylation, even in the presence of sufficient oxygen. This phenomenon allows cancer cells to produce energy rapidly and generate metabolic intermediates necessary for proliferation. Increased glucose uptake and conversion to lactate leads to lactate accumulation even under aerobic conditions. It enables support for anabolic processes, such as nucleotide and lipid synthesis, through the diversion of glycolytic intermediates [7].

Role of Hypoxia-Inducible Factor 1-Alpha in Metabolic Reprogramming

HIF-1α is activated under hypoxic conditions and induces the expression of various genes involved in metabolic reprogramming. Through its influence, HIF-1α facilitates the adaptation of cancer cells to low oxygen and nutrient availability. Upregulation of glycolytic enzymes enhances the glycolytic pathway. Induction of glucose transporters, such as GLUT1, increases glucose uptake [2].

Key Metabolic Pathways in Cancer

Glycolysis: Metabolic alterations are common in cancer cells that reallocate resources toward glycolysis to meet the high energy demands and to provide precursors for biosynthesis. Some of the key enzymes involved in glycolysis including hexokinase, phosphofructokinase, and pyruvate kinase are upregulated in these cells. These changes in the glycolytic rates facilitate fast cell growth and division. The preference for glycolysis is a characteristic of the cancer cells known as the Warburg effect [27].

Lipid metabolism: Lipid metabolism, particularly the synthesis and expenditure of fatty acids, is usually dysregulated in cancer cells. HIF-1α plays a role in influencing enzymes that participate in lipid synthesis, essential for the formulation of membranes and energy production. This metabolic change is beneficial to the cells of a tumor. Better lipid metabolism is another key to the cancer cell’s ability to support it in different conditions [28].

Amino acid metabolism: Tumor cells have definite preferences for specific amino acids that they require for their functions of growth and multiplication. For example, increased rates of glutamine breakdown provide nitrogen and carbon which is crucial for nucleotide synthesis and energy production. This reliance on amino acids furthers the biosynthesis necessary for the propagation of the tumor. The use of amino acids is an essential feature of cancer cell metabolism [29].

Pentose phosphate pathway: The pentose phosphate pathway is important in the biosynthesis of NADPH and other essential molecules such as ribose-5-phosphate that are required by the various anabolic pathways present in the body. Stimulation of HIF-1α increases the flow through this route leading to increased biosynthesis and antioxidant activity. This pathway caters to the metabolic needs of the highly proliferative cancer cells. It is also important in the regulation of redox reactions and the supply of components necessary for the growth of tumors [30].

Acidosis and Tumor Microenvironment

The reliance on glycolysis results in increased lactate production, leading to acidosis in the tumor microenvironment. This acidic environment can promote tumor invasion and immune evasion while further influencing metabolic adaptations in cancer cells. Promotion of cancer cell invasiveness and suppression of effective immune responses in the tumor microenvironment also occur [31].

Metabolic Flexibility

Cancer cells exhibit remarkable metabolic flexibility, allowing them to switch between different energy sources based on nutrient availability and environmental conditions. This adaptability is crucial for tumor survival and progression. Switching from glycolysis to oxidative phosphorylation when oxygen levels improve or nutrients are available. Utilization of alternative substrates, such as fatty acids or ketone bodies, during nutrient deprivation [32].

Cell survival and invasion in cancer

Cell survival and invasion are critical processes that underpin cancer progression and metastasis. HIF-1α plays a pivotal role in regulating these processes, enabling cancer cells to adapt to adverse microenvironments and promoting tumor aggressiveness. Understanding the mechanisms involved in cell survival and invasion is essential for developing effective cancer therapies [10].

Mechanisms of Cell Survival

**Figure 4:**
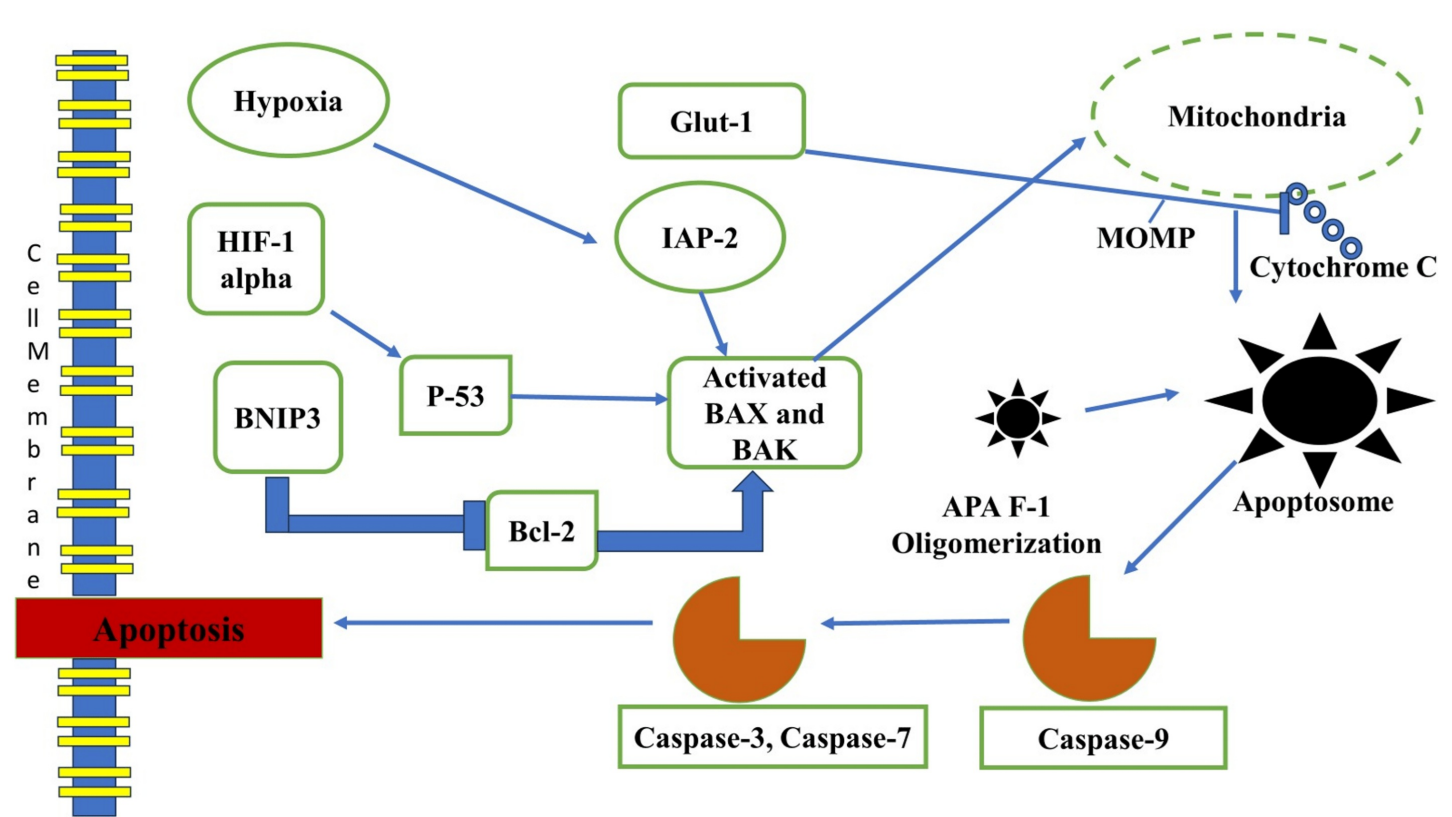
Autophagy often serves as a survival mechanism against toxicity from xenobiotic exposure. APA F-1: apoptotic protease activating factor 1; Caspase-3: cysteine-aspartic protease 3; Bcl-2: B-cell lymphoma 2; P-53: protein 53; BNIP3: Bcl-2/adenovirus E1B 19 kDa-interacting protein 3; GLUT-1: glucose transporter 1; IAP-2: inhibitor of apoptosis protein 2; BAX: Bcl-2-associated X protein; BAK: Bcl-2 homologous antagonist/killer; MOMP: mitochondrial outer membrane permeabilization; MOMP: mitochondrial outer membrane permeabilization Image credit: Sana Qausain

Cancer cells often encounter stressful conditions, such as low oxygen levels, nutrient deprivation, and exposure to therapeutic agents (Figure 4). HIF-1α mediates several survival mechanisms that allow cancer cells to withstand these challenges.

Upregulation of anti-apoptotic proteins: HIF-1α promotes the expression of anti-apoptotic proteins, which inhibit programmed cell death and enhance cell survival. B-cell lymphoma 2 (Bcl-2) inhibits apoptosis by preventing mitochondrial outer membrane permeabilization and is involved in cell cycle regulation [2].

Metabolic adaptations: HIF-1α engulfs metabolic adaptation in cancer cells where the ability of producing energy through glycolysis in hypoxic conditions is enhanced. This shift helps the cells to survive in conditions of low oxygen and supplies the cell with the metabolite that paves the way for rapid utilization. Cancer cells can meet their energy requirements through glycolysis even in the absence of oxygen, and in the presence of oxygen, oxidative phosphorylation is much lower than in normal cells. It is vital for the conservation of tumor growth and proliferation with metastasis to other regions of the body [33].

Autophagy: It has been reported that HIF-1α can activate autophagy, a process in which degradative cellular components such as organelles and proteins in stressed cells enhance cell survival. As autophagy enables cancer cells to continuously regulate their nutrient intake to sustain their normal activity as well as survive in conditions of nutrient scarcity, autophagy is important to cancer cells. Autophagy, therefore, rallies for the recycling of cellular components for the sustenance and perpetuity of cancer cells. This adaptive mechanism is crucial in the regulation of cell stress and the survival of tumors [34].

Mechanisms of Invasion

**Figure 5:**
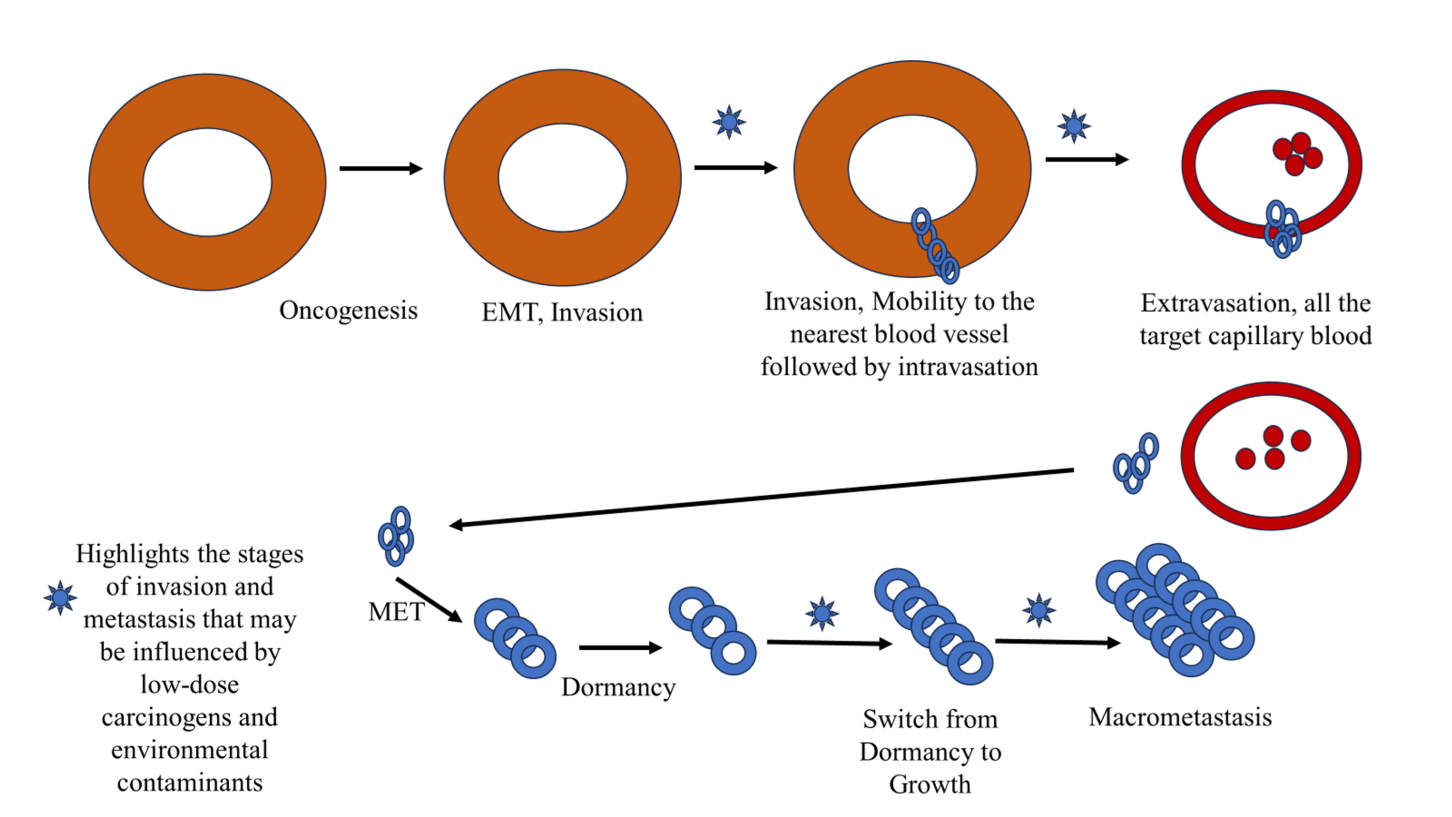
Key stages of cancer invasion and metastasis. EMT: epithelial-mesenchymal transition; MET: mesenchymal-epithelial transition Image credit: Mohd. Basheeruddin

Invasion is a crucial step in cancer metastasis, allowing tumor cells to breach surrounding tissues and enter the bloodstream or lymphatic system. HIF-1α drives several key mechanisms involved in the invasion of cancer cells (Figure 5).

Epithelial-mesenchymal transition (EMT): The EMT, which is facilitated by HIF-1α, increases the migratory and invasive potential of cancer cells. Tumor cell invasion is facilitated by EMT, which is defined by the acquisition of mesenchymal characteristics and the loss of epithelial markers. EMT is driven by transcription factors including Twist and Snail, whose expression is induced by HIF-1α [35].

Matrix metalloproteinases: MMPs are enzymes that break down ECM components, and HIF-1α increases their expression. The invasion and spread of cancer cells depend on this breakdown. MMPs encourage the ECM to break down, which lets tumor cells infiltrate neighboring tissues [22].

Increased motility: HIF-1α enhances cancer cell migration and invasion by changing cytoarchitecture remodeling and changes in migration-related proteins. This greatly helps cancer cells by increasing their motility so that they can easily invade other tissues. In fixing the cytoskeleton of the cell, HIF-1α promotes the migration required for metastasis. The disordered motility is noted to be a strong proponent of the aggressive nature of the cancer [35].

Chemotaxis: Lacking oxygen the cancer cells release several chemokines and growth factors that help them navigate to areas within the tumor with less oxygen or more nutrients. One of the ways in which this is achieved is through the movement of cancer cells toward locations that may better suit their requirements This movement is referred to as chemotaxis. The possibility of approaching these regions benefits tumor survival and growth [36].

Interaction With the Tumor Microenvironment

The tumor microenvironment plays a significant role in mediating cell survival and invasion. Hypoxia alters the composition of the tumor microenvironment, influencing both cancer cell behavior and the surrounding stromal cells.

Tumor-associated macrophages (TAMs): Several studies have illustrated that under hypoxic conditions, TAMs can acquire a pro-tumorigenic phenotype and reflect several factors that can sustain cell survival and invasion. HIF-1α plays a part in the modulation of the polarization and activation of these macrophages. This polarization enhances tumor growth and metastasis as they are an important support factor of cancer. TAMs play a very crucial role in the tumor microenvironment and its development [37].

Extracellular matrix remodeling: Focusing on the level of cancer cells and the surrounding host environment, one of the most critical processes is the invasion of the cancer cells into the ECM. HIF-1α is also involved in the expression of enzymes such as MMPs involved in the remodeling of ECM. This remodeling process facilitates the invasion of tumor cells to the surrounding tissues much easier. Appropriate modification of ECM and its composition are the determining factors of tumor cell migration and metastasis [38].

Regulation of hypoxia-inducible factor 1-alpha

HIF-1α is an essential transcription factor that controls how cells react to low oxygen concentrations or hypoxia. Its activity is strictly regulated by several mechanisms that guarantee proper cellular response to variations in oxygen availability. Understanding the regulation of HIF-1α is essential for developing therapeutic strategies targeting hypoxia-related diseases, including cancer [11].

Oxygen-Dependent Regulation

Hydroxylation: PHDs hydroxylate HIF-1α on particular proline residues under normoxic circumstances. Its identification by the VHL tumor suppressor protein is facilitated by this post-translational alteration, which results in proteasomal destruction. PHDs, namely, PHD1, PHD2, and PHD3, are important enzymes that are involved. Hydroxylation marks HIF-1α for degradation, preventing its accumulation in oxygen-rich environments [39].

Interaction with von Hippel-Lindau: The E3 ubiquitin ligase complex includes the VHL protein, which directs the breakdown of hydroxylated HIF-1α. Hypoxic conditions prevent hydroxylation, which prevents VHL from binding to HIF-1α and causes it to stabilize. This stabilization enables HIF-1α to transactivate its target genes. It is invaluable to understand how VHL controls hypoxia-inducible factors, particularly HIF-1α, to better understand the cellular response [40].

Non-oxygen-Dependent Regulation

HIF-1α can also be regulated by various factors independent of oxygen levels, allowing it to respond to different cellular stresses.

Growth factors: Growth factors such as VEGF and EGF can initiate signaling pathways that maintain HIF-1α stability, even in normoxic environments. The pathways that support HIF-1α stability and activity are PI3K/Akt and MAPK [41].

Oncogenes and tumor suppressors: Some oncogenes, such as the ones seen in *KRAS* or *MYC*, help increase the level of HIF-1α, thereby contributing to tumor development. On the other hand, target genes that have tumor suppressor proteins such as *p53* can regulate HIF-1α in various ways. Tarrado-Castellarnau et al. decided to investigate how these interactions affect the HIF-1α regulation balance and change cancer cell behavior. The mechanism of action, interaction between oncogenes and tumor suppressors, as well as HIF-1α, is very important in terms of tumorigenesis and chemotherapy sensitivity [42].

Feedback Mechanisms

HIF-1α activity is subject to feedback regulation, whereby its target genes can influence HIF-1α levels and activity in a looped manner. For example, increased expression of VEGF can promote angiogenesis but also lead to changes in oxygen supply, further modulating HIF-1α activity [6].

Therapeutic approach

HIF-1α is an important player in cancer progression because it promotes angiogenesis, metabolic reprogramming, and cell survival in low-oxygen environments. A potential therapeutic approach to stop tumor growth and enhance treatment results is to target HIF-1α. Here, we explore various approaches for the therapeutic targeting of HIF-1α [2].

Direct Inhibition of Hypoxia-Inducible Factor 1-Alpha

Small molecule inhibitors: Several tiny compounds have been created that specifically block HIF-1α action. These substances can prevent HIF-1α from binding to DNA or dimerizing with HIF-1β. PT2385 is a selective inhibitor that disrupts the HIF-1α pathway and has shown efficacy in preclinical models. EZN-2208 is an antisense oligonucleotide that targets HIF-1α mRNA.

Peptide-based inhibitors: Small-molecule peptides that prevent the binding of HIF-1α to HIF-1β, or prevent the DNA binding ability of HIF-1α, are currently under investigation. These inhibitors intend target the inhibition of HIF-1α, which is important for tumor growth and proliferation. Thus, these peptide-protein interactions could potentially be addressed by using peptide-based inhibitors for the HIF-1α-dependent cancers. Their efficacy and possible uses in clinical practice are still under investigations [43].

Targeting Hypoxia-Inducible Factor 1-Alpha Regulation

Modulating prolyl hydroxylases: Increasing the activity of PHDs makes it possible to degrade HIF-1α even under hypoxic conditions, as discussed above. However, some agents antagonize the action of HIF-PH and increase the levels of PHD stabilized from HIF-1α. The idea behind this approach is to capitalize on the inherent processes of degradation in controlling HuWE1 and other HIF-1α-dependent pathways. Reducing HIF-1α accumulation may also be therapeutically targeted through PHDs, which are associated with identified PHDs.

VHL pathway activation: The level of HIF-1α can be reduced by deactivating the VHL tumor suppressor pathway. Some of the therapeutic approaches may include employing reagents that fix the VHL dysfunction in tumors that are characterized by a dysfunctional VHL. This restoration is very efficient at reducing HIF-1α levels and negating the oncogenic impact of the same. Hence, targeting the VHL pathway appears to be a useful strategy for controlling tumorigenesis associated with HIF-1α [40].

Targeting Hypoxia-Inducible Factor 1-Alpha Downstream Effects

Inhibiting angiogenesis: Modulation of these downstream signals can be beneficial as HIF-1α upregulates the levels of chemicals that promote angiogenesis, such as VEGF. A class of medications called anti-VEGFs such as bevacizumab can block the effects of HIF-1α-induced angiogenesis. Among these is the inhibition of the synthesis of a protein known as VEGF, which aids in the development of new blood vessels that the tumor requires. As a result, this strategy helps to lower the tumor’s growth rate and capacity to support both the growth and production of metastases.

Inhibiting glycolytic pathways: Some glycolytic enzymes upregulated by HIF-1α might be targeted to hamper cancer cell adaptation to a hypoxic environment. Some of the glycolytic metabolites can act as allosteric inhibitors of key enzymes in the glycolytic pathway such as hexokinase or pyruvate kinase and thereby decrease the glycolytic flux in cancer cells. This reduction chokes off the energy and metabolites that are imperative for tumor growth. As disrupting glycolysis, all these inhibitors target the critical element of the metabolism in cancer cells that is regulated by HIF-1α [18].

Combination Therapies

HIF-1α inhibitors can boost the total outcome by using it in combination. Combination therapies could be more effective in carrying out a variety of actions on tumors and, thus, could be more effective than single-agent treatments. They use synergistic features of further amendments to HIF-1α inhibition and a particular therapeutic approach. Sometimes integration of therapies can be more effective in fighting cancer than using one therapy alone.

Chemotherapy and radiotherapy: HIF-1α antagonism can enhance the tumor’s response to chemotherapy and radiotherapy, as it compromises cell survival signaling. This makes cancer cells more susceptible to the kind of treatments that conventional medicine has to offer. HIF-1α inhibitors are combined with conventional chemotherapy drugs or radiation to enhance the outcome of the therapy. This strategy has the goal of increasing the optimization of the available treatments and the quality of treatment results [10].

Immunotherapy: Targeting HIF-1α can indeed modify the tumor microenvironment with the possible enhancement of immunotherapeutics. HIF-1α inhibitors affect the conditions in the tumor microenvironment and thereby improve the ability of the host immune system to perceive and destroy cancer cells. This combination approach has been designed to enhance the general outcome of immunotherapy. Interfering with the tumor microenvironment should increase the activity of immunotherapy with refractory tumors [37].

Novel Delivery Systems

Designing strategies for targeting HIF-1α inhibitors can potentially enhance the general effectiveness of treatment by selectively delivering the treatment directly to the malignant cells. This approach decreases the deleterious effects which are observed more distally and these effects contribute to lower systemic toxicity. In addition, these systems also improve the efficacy of the drug as the inhibitors are effectively delivered to the region of the tumor. Cancer treatment has been designed to ensure the delivery of the treatment to the diseased cells only while sparing the other healthy tissues [3].

Nanoparticles: Conventional methods of managing HIF-1α are invasive and systemically toxic but nanotechnology can deliver HIF-1α inhibitors directly to the site of tumor, thereby increasing drug availability and minimizing side effects. One tactic for improving the indirect treatment of inhibitors is the encapsulation of these enhancers within nanoparticles to increase their point of concentration at the tumor while minimizing damage to the rest of the body. It is hoped that such specific targeting will enhance the efficacy of treatments and reduce the side effects that may be associated with therapies. The inhibitors can be delivered in specific sites and released systematically because of nanoparticles [44].

RNA interference: A new approach is the deliberate targeting of HIF-1α with small interfering RNA (siRNA) or antisense oligonucleotides. These RNA-mimicking methodologies can effectively intervene with the spurt of HIF-1α in the process of tumor development. RNA interference is specific and, when applied genetically to target the HIF-1α pathway, has the propensity of offering the necessary control. It has the capability of increasing the treatment’s effectiveness and at the same time minimizing side effects [45].

Small molecule inhibitors

Small molecule inhibitors targeting HIF-1α are a promising strategy in cancer therapy. These compounds can interfere with HIF-1α's activity, stability, or interaction with other proteins, thereby inhibiting tumor growth and metastasis. Below are some key small molecule inhibitors currently under investigation or in clinical use [10].

Direct Hypoxia-Inducible Factor 1-Alpha Inhibitors

PT2385: PT2385 inhibits only HIF-1α by binding to it and preventing its binding to HIF-1β, thus preventing it from initiating any transcription. This targeted approach has shown a possibility of treating other cancers such as clear-cell renal cell carcinoma. The molecular mechanism underlying this idea is that by regulating the ability of HIF-1α to bind to its partner HIF-1β, PT2385 can inhibit tumor growth. They make up a promising approach to cancer treatment [46].

EZN-2208: EZN-2208 is an antisense oligonucleotide used to inhibit the HIF-1α mRNA in a specific manner. It has been investigated in experimental models for its efficiency on solid tumors. This, in turn, reduces HIF-1α levels, thus making EZN-2208 potentially useful in the treatment of tumor growth and progression. Its uniqueness is in its targeted approach presents a new way of treating cancer [47].

Acriflavine: Acriflavine initially used as an antimalarial and antiseptic compound antagonizes HIF-1α by blocking its ability to bind to DNA. Studies have been conducted out on this compound concerning its anticancer efficacy. Acriflavine’s potential in helping control cancer arises from its inhibition of HIF-1α’s DNA-binding capabilities. The somewhat dual nature of the agent suggests its applicability for therapeutic uses [48].

Hypoxia-Inducible Factor 1-Alpha Stabilization Inhibitors

2-Methoxyestradiol: VE-827 inhibits 2-methoxyestradiol-mediated degradation of HIF-1α by preventing its binding to coactivators and stabilizes the HIF-1α leading to its nuclear accumulation. This approach is at present being researched for its ability to affect cancer cells and has been tested in numerous types of cancer. Thus 2-methoxyestradiol is designed to disrupt the binding and stability of HIF-1α and in this way disrupt tumor growth. It has been under research concerning its capability of being an anticancer agent [49].

Dimethyloxalylglycine (DMOG): DMOG is considered to be an inhibitor of PHD through which HIF-1α is prevented from being hydroxylated. This leads to the accumulation of HIF-1α and improved cell signaling pathways to hypoxia. Due to its observed antioxidant activity and potential to influence conditions related to low oxygen levels, DMOG is being considered for affecting hypoxia and cellular adaptation to the same. Little is known about its workings in cancer and other diseases [50].

Hypoxia-Inducible Factor 1-Alpha Modulators

Cobalt(II) chloride (CoCl₂): CoCl₂ can copy hypoxic conditions by blocking PHD enzymes, which, in turn, stabilizes HIF-1α. This stabilization increases HIF-1α levels and is applied in research for analyzing cell reactions to hypoxia. CoCl₂ has been used as an artificial hypoxia model. It can help reveal the impact of low oxygen on cellular processes.

Rhein: Rhein also suppresses the stabilized HIF-1α at the protein level and its transcriptional activity, through proteasome degradation. This compound has been studied in animal and human tumor tissues and n numerous cancer types. Through the inhibition of function and stability of HIF-1α, Rhein has a therapeutic effect. Its being selective for HIF-1α makes it a potent candidate for anticancer activity [51].

Inhibitors Targeting Hypoxia-Inducible Factor 1-Alpha Signaling Pathways

Sorafenib: Sorafenib is a small molecule that has multiple kinase profiles to inhibit tumor cell proliferation and has features of HIF-1α suppression. It is applied in the therapy of renal cell carcinoma and hepatocellular carcinoma. Through the inhibition of important signaling intermediates, sorafenib can inhibit cellular proliferation and the hypoxic response. Because of its biphasic effect, it is used as a therapeutic agent in oncology [52].

Sunitinib: Sunitinib acts as an antiangiogenic agent by interfering with multiple receptor tyrosine kinases, resulting in decreased synthesis of VEGF and enhanced HIF-1α. This compound is approved for use in the management of renal cell carcinoma and gastrointestinal stromal tumors. Thus, sunitinib is designed to inhibit the key angiogenic factors and affect tumor vessel formation and growth. Its ability to control these cancers is the reason why it is considered to have therapeutic benefits [53].

Combination Approaches

HIF-1α inhibitors with chemotherapy: HIF-1α inhibitors such as PT2385 when used in conjunction with conventional chemotherapy improve the effectiveness of the treatment on tumors. This combination was designed to enhance oxygenation status as well as to target otherwise conventional academic therapeutic strategies. The interaction may lead to enhanced effects in the treatment of cancer. Combining these inhibitors with chemotherapy may help in eradicating the resistant cancer cells and subsequently enhance treatment outcomes.

HIF-1α inhibitors with immunotherapy: HIF-1α inhibitors could also be synergized with immune checkpoint inhibitors to increase immune control of tumors. This strategy focuses on improving immunotherapy by altering the environment of the tumor to encourage immune response. Together, it may enhance the immune response to recognize and destroy malignant cells. This approach can be seen as a possible modernization of the methods for increasing the effectiveness of immunotherapeutic interventions [10].

RNA interference and its application in targeting hypoxia-inducible factor 1-alpha

RNA interference (RNAi) is a powerful biological process used to silence gene expression at the post-transcriptional level. This mechanism has been harnessed for therapeutic applications, particularly in targeting HIF-1α, a key regulator in cancer and other diseases associated with hypoxia.

Mechanism of RNA Interference

Small interfering RNAs (siRNAs): Small interfering RNAs are double-stranded RNA molecules that are usually 21-23 nucleotides long (siRNAs). When complementary mRNA sequences attach to siRNAs, the RNA-induced silencing complex (RISC) degrades the mRNAs.

MicroRNAs (miRNAs): MicroRNAs or miRNAs are short RNA chains that do not code in proteins but act as modulators of gene expression by inhibiting the translation of their target mRNAs or by stabilizing mRNA molecules in a degradation pathway. The following miRNAs may, therefore, control HIF-1α levels and consequently its target signaling pathways. Depending on miRNAs’ contribution to the regulation of HIF-1α levels, they contribute to multiple processes and pathologies. This regulatory function makes them pertinent candidates for therapeutic intervention as well as biomarkers [45].

Targeting Hypoxia-Inducible Factor 1-Alpha With RNA Interference

siRNA targeting HIF-1α: Specific siRNAs can be designed to target and degrade the mRNA of HIF-1α, effectively reducing its expression and activity in cancer cells. This approach has demonstrated promise across various cancer types, resulting in reduced tumor growth and increased sensitivity to therapies. By specifically targeting HIF-1α, siRNAs offer a precise method for managing cancer and improving treatment outcomes.

miRNA modulation: Certain miRNAs can directly target HIF-1α or its downstream effectors, impacting cancer cell proliferation and survival. For example, miR-155 is associated with promoting HIF-1α expression, and targeting it can reduce HIF-1α levels. Similarly, miR-210 is often upregulated in hypoxic conditions and can reinforce HIF-1α signaling [54].

Delivery Methods for RNA Interference

The delivery of RNAi molecules is an important factor for the success of RNAi therapeutics. To enhance this process, various delivery systems are still under investigation. These include lipid nanoparticles for systemic administration, viral vectors for efficient transduction, and polymeric systems for controlled release. Both of them are designed to improve RNAi stability and incorporation into cells.

Lipid nanoparticles: Lipid nanoparticles are biocompatible, and the encapsulated RNA molecules can be internalized into the cell through endocytosis. These carriers are good for systemic application and shield RNA from degradation. Due to their effectiveness in the delivery of RNA, they are applicable in therapeutic activities [55].

Viral vectors: In this method, viral vectors are also designed and employed to introduce siRNA or miRNA sequences into particular cells. They offer high TT operation and simplicity besides yielding stable splicing of RNAi molecules. That they are efficient in gene delivery makes them applicable in therapeutic contexts.

Polymeric carriers: Biodegradable polymeric carriers bear and transport RNA molecules and target particular tissues. Such polymers can be formulated in a way that they are capable of releasing the drug in a controlled manner and are stable at the same time. The manner in which these particles target tissues and how they can modulate release kinetics boosts RNA medicinal chemistry [56].

Therapeutic Applications

Cancer therapy: HIF-1α levels, if reduced, have been seen to decrease tumor growth and metastasis by a large percentage. This reduction also increases the rate of cancer cell killing by radiotherapy and chemotherapy, thus improving the success rate of the treatment. HIF-1α is a known factor, and when therapies interfere with the same, the tumor possibilities of adapting to hypoxic situations are curbed, limiting cancer growth. It is expected it will help in enhancing patient outcomes of different cancer types in the future.

Ischemic diseases: HIF 1α may be viewed as an ideal therapeutic target in ischemic heart disease, as it modulates angiogenetic responses and enhances cell survival. Altering HIF-1α has the potential to facilitate improved angiogenesis and promote tissue regeneration under ischemia. This strategy is to enhance the heart function in ischemic heart disease patients to effect recovery. Thus, the modulation of these pathways can, in principle, be addressed via targeted treatments that would enhance the prognosis of such conditions [2].

Challenges and Considerations

Off-target effects: siRNAs can potentially act on other genes, thereby causing other ailments or side effects. This problem has been solved by careful selection of siRNAs against their targets such that the effects will not be off-target.

Delivery efficiency: The delivery of small RNAi molecules to the target tissue is still a problem despite constant research on the same. The problem to address is the lack of advanced delivery systems to address issues of cellular uptake and stability to improve RNAi therapies.

Immune response: RNAi molecules can provoke immune reaction, which can lower its usefulness in treatments. To increase the stability and effectiveness of RNAi constructs, it is important to alter the RNAi constructs so that they cannot be detected by the immune system [50].

Immunotherapy approaches

Immunotherapy has emerged as a promising strategy in cancer treatment, leveraging the immune system to target and eliminate cancer cells. Targeting HIF-1α can enhance the efficacy of immunotherapy by modulating the tumor microenvironment and improving immune responses [57]. Table 1 presents key immunotherapy approaches that can be used in conjunction with HIF-1α targeting [57].

**Table 1:** Key immunotherapy approaches that can be combined with HIF-1α targeting include checkpoint inhibitors, CAR T-cell therapy, and cancer vaccines. HIF-1α: hypoxia-inducible factor 1-alpha; CAR: chimeric antigen receptor; PD-1: programmed cell death protein 1; PD-L1: programmed death-ligand 1; TIL: tumor-infiltrating lymphocyte; T-VEC: talimogene laherparepvec Table credit: Sana Qausain

Immunotherapy approach	Mechanism	Synergy with HIF-1α targeting
Immune checkpoint inhibitors	PD-1/PD-L1 inhibitors enhance T cells	HIF-1α and PD-1/PD-L1 are reported to improve the response
Cancer vaccines	Therapeutic vaccines boost tumor immunity	It also substantiates the finding that inhibition of HIF-1α enhances antigen presentation
Adoptive cell transfer	Both CAR T cells and TILs focus on cancers	Reducing HIF-1α improves TIL function
Oncolytic virus therapy	T-VEC viruses kill tumors and enhance immunity	Increased oncolytic effectiveness of Ada-Hudson oncolytic virus through targeting HIF-1α
Combination therapies	The effectiveness of the therapy increases when HIF-1α inhibitors are used	The current studies also show that HIF-1α inactivation enhances immune infiltration
Targeting the tumor microenvironment	Alteration of the microenvironment enhances immunity	HIF-1α targeting improves the general outcomes of the therapies

Immune Checkpoint Inhibitors

Mechanism: Immune checkpoint drugs increase T-cell activation and proliferation against malignancies by blocking proteins that impede immune responses. In the tumor microenvironment, programmed cell death protein 1 (PD-1) inhibitors such as pembrolizumab and nivolumab can revitalize worn-out T cells. Atezolizumab and durvalumab are examples of programmed death-ligand 1 (PD-L1) inhibitors that stop tumor cells from eluding immune recognition.

Synergy with hypoxia-inducible factor 1-alpha targeting: When targeting HIF-1α, this may be responsible for alterations of PD-L1 including immune evasion. Suppression of HIF-1α might improve the activity of PD-1/PD-L1 inhibitors because other immune cells are more likely to act synergistically with them. The idea of employing both active and passive immune responses might potentiate the action of immunotherapy against tumors and complement the existing treatment regimens [58].

Cancer Vaccines

Mechanism: Cancer vaccines activate the immune system to identify and combat antigens unique to tumors. Therapeutic vaccinations aim to improve the immune system’s ability to fight tumor antigens, thereby treating malignancies that have already spread. Vaccines containing dendritic cells loaded with tumor antigens stimulate T lymphocytes.

Synergy with hypoxia-inducible factor 1-alpha targeting: HIF-1α can be suppressed to enhance the expression of tumor antigen and the maturation of dendritic cells. This synergistic effect increases the potential of cancer vaccines as they help the immunological system respond to the tumor. Thus, it has been found that the use of HIF-1α inhibitors in conjunction with cancer vaccines will result in better tumor recognition and destruction by the immune system [2].

Adoptive Cell Transfer

Mechanism: To improve a patient’s immunity against tumors, activated T cells or other immune cells are infused into them by adoptive cell transfer. Through genetic engineering, T cells are made to express receptors that specifically target tumor antigens to facilitate chimeric antigen receptor (CAR) T-cell therapy. Tumor-infiltrating lymphocyte (TIL) therapy involves the expansion and reintroduction of T lymphocytes obtained from tumor samples back into the patient.

Synergy with hypoxia-inducible factor 1-alpha targeting: Reducing HIF-1α expression could promote TIL function and survival in the context of hypoxia within the tumor microenvironment. This improvement enhances the output of adoptive cell therapies in a way that TIL functionality against cancer cells is enhanced. Reduction of HIF-1α assists in overcoming the hypoxic problems; therefore, improving the therapeutic gains [59].

Oncolytic Virus Therapy

Mechanism: Oncolytic viruses are those that are capable of infecting and killing cancer cells and concurrently provoking an immune response against the tumor. Talimogene laherparepvec (T-VEC) is a singly mutated herpes simplex virus that promotes both direct local destruction of tumor cells and immune stimulation in the body.

Synergy with hypoxia-inducible factor 1-alpha targeting: HIF-1α can be targeted to promote antitumor immunity and reduce immunosuppressive elements to increase the effect of oncolytic viruses. This combined modality can result in enhanced tumor eradication and an armaggedon-like attack on the tumor cells [60].

Combination Therapies

Mechanism: It is possible to improve treatment efficacy and get past resistance mechanisms by combining various therapy methods. Immunological checkpoint inhibitors and HIF-1α inhibitors together improve antitumor immunity and decrease immunological evasion. Additionally, combining HIF-1α inhibitors with chemotherapy or radiotherapy improves the immune response and sensitizes tumors to conventional therapies.

Synergy with hypoxia-inducible factor 1-alpha targeting: Inhibition of HIF-1α decreases tumor hypoxia and improves immune cell infiltration, increasing the efficacy of the combined treatments. It enhances the prognosis by providing a suitable background for therapeutic products and immune responses [61].

Targeting the Tumor Microenvironment

Mechanism: Modulating the tumor microenvironment can enhance immune responses and improve therapeutic outcomes. Inhibiting immunosuppressive cells involves targeting regulatory T cells or tumor-associated macrophages that promote immune suppression. Enhancing cytokine production can be achieved by using agents that stimulate the production of pro-inflammatory cytokines to promote antitumor immunity.

Synergy with hypoxia-inducible factor 1-alpha targeting: Targeting HIF-1α can alter the immune environment in a way that decreases the immunosuppressive cell population and enhances the effectiveness of other immunotherapies. The use of this strategy improves the general cellular immunity against tumors, resulting in better therapeutic results [62].

## Conclusions

A promising therapeutic approach for treating cancer involves focusing on HIF-1α, which influences the tumor microenvironment and boosts immune responses. Angiogenesis, metabolic tolerance to low oxygen levels, and tumor survival are all significantly impacted by HIF-1α. Researchers can efficiently decrease HIF-1α activity and its related pathways by using a variety of strategies, including immunotherapy, RNAi, and small molecule inhibitors. This will enhance treatment outcomes and promote the sensitivity of currently available medicines. There is great promise for bypassing resistance mechanisms and boosting antitumor immunity when HIF-1α targeting is combined with immunotherapeutic approaches such as immune checkpoint inhibitors and cancer vaccinations. As the understanding of HIF-1α’s role in cancer biology expands, further investigation into innovative combination therapies and delivery methods will be essential to optimize patient outcomes and advance the field of oncology.

## References

[1] Chen G, Wu K, Li H, Xia D, He T (2022). Role of hypoxia in the tumor microenvironment and targeted therapy. Front Oncol.

[2] Infantino V, Santarsiero A, Convertini P, Todisco S, Iacobazzi V (2021). Cancer cell metabolism in hypoxia: role of HIF-1 as key regulator and therapeutic target. Int J Mol Sci.

[3] Onnis B, Rapisarda A, Melillo G (2009). Development of HIF-1 inhibitors for cancer therapy. J Cell Mol Med.

[4] Muz B, de la Puente P, Azab F, Azab AK (2015). The role of hypoxia in cancer progression, angiogenesis, metastasis, and resistance to therapy. Hypoxia (Auckl).

[5] Lugano R, Ramachandran M, Dimberg A (2020). Tumor angiogenesis: causes, consequences, challenges and opportunities. Cell Mol Life Sci.

[6] Krock BL, Skuli N, Simon MC (2011). Hypoxia-induced angiogenesis: good and evil. Genes Cancer.

[7] Liberti MV, Locasale JW (2016). The Warburg effect: how does it benefit cancer cells?. Trends Biochem Sci.

[8] Siemann DW, Horsman MR (2015). Modulation of the tumor vasculature and oxygenation to improve therapy. Pharmacol Ther.

[9] Rofstad EK, Galappathi K, Mathiesen BS (2014). Tumor interstitial fluid pressure-a link between tumor hypoxia, microvascular density, and lymph node metastasis. Neoplasia.

[10] Bui BP, Nguyen PL, Lee K, Cho J (2022). Hypoxia-inducible factor-1: a novel therapeutic target for the management of cancer, drug resistance, and cancer-related pain. Cancers (Basel).

[11] Ziello JE, Jovin IS, Huang Y (2007). Hypoxia-inducible factor (HIF)-1 regulatory pathway and its potential for therapeutic intervention in malignancy and ischemia. Yale J Biol Med.

[12] Gaete D, Rodriguez D, Watts D, Sormendi S, Chavakis T, Wielockx B (2021). HIF-prolyl hydroxylase domain proteins (PHDs) in cancer-potential targets for anti-tumor therapy?. Cancers (Basel).

[13] Magar AG, Morya VK, Kwak MK, Oh JU, Noh KC (2024). A molecular perspective on HIF-1α and angiogenic stimulator networks and their role in solid tumors: an update. Int J Mol Sci.

[14] Shibuya M (2011). Vascular endothelial growth factor (VEGF) and its receptor (VEGFR) signaling in angiogenesis: a crucial target for anti- and pro-angiogenic therapies. Genes Cancer.

[15] Raica M, Cimpean AM (2010). Platelet-derived growth factor (PDGF)/PDGF receptors (PDGFR) axis as target for antitumor and antiangiogenic therapy. Pharmaceuticals (Basel).

[16] Hu B, Cheng SY (2009). Angiopoietin-2: development of inhibitors for cancer therapy. Curr Oncol Rep.

[17] Farooq M, Khan AW, Kim MS, Choi S (2021). The role of fibroblast growth factor (FGF) signaling in tissue repair and regeneration. Cells.

[18] Niu G, Chen X (2010). Vascular endothelial growth factor as an anti-angiogenic target for cancer therapy. Curr Drug Targets.

[19] He Y, Sun MM, Zhang GG, Yang J, Chen KS, Xu WW, Li B (2021). Targeting PI3K/Akt signal transduction for cancer therapy. Signal Transduct Target Ther.

[20] Morrison DK (2012). MAP kinase pathways. Cold Spring Harb Perspect Biol.

[21] Wang X, Khalil RA (2018). Matrix metalloproteinases, vascular remodeling, and vascular disease. Adv Pharmacol.

[22] Cabral-Pacheco GA, Garza-Veloz I, Castruita-De la Rosa C (2020). The roles of matrix metalloproteinases and their inhibitors in human diseases. Int J Mol Sci.

[23] Cai L, Xiong X, Kong X, Xie J (2017). The role of the lysyl oxidases in tissue repair and remodeling: a concise review. Tissue Eng Regen Med.

[24] Michaelis UR (2014). Mechanisms of endothelial cell migration. Cell Mol Life Sci.

[25] Darland DC, D'Amore PA (1999). Blood vessel maturation: vascular development comes of age. J Clin Invest.

[26] Navarro C, Ortega Á, Santeliz R (2022). Metabolic reprogramming in cancer cells: emerging molecular mechanisms and novel therapeutic approaches. Pharmaceutics.

[27] Alfarouk KO, Verduzco D, Rauch C (2014). Glycolysis, tumor metabolism, cancer growth and dissemination. A new pH-based etiopathogenic perspective and therapeutic approach to an old cancer question. Oncoscience.

[28] Mylonis I, Simos G, Paraskeva E (2019). Hypoxia-inducible factors and the regulation of lipid metabolism. Cells.

[29] Jin J, Byun JK, Choi YK, Park KG (2023). Targeting glutamine metabolism as a therapeutic strategy for cancer. Exp Mol Med.

[30] Stincone A, Prigione A, Cramer T (2015). The return of metabolism: biochemistry and physiology of the pentose phosphate pathway. Biol Rev Camb Philos Soc.

[31] de la Cruz-López KG, Castro-Muñoz LJ, Reyes-Hernández DO, García-Carrancá A, Manzo-Merino J (2019). Lactate in the regulation of tumor microenvironment and therapeutic approaches. Front Oncol.

[32] Kreuzaler P, Panina Y, Segal J, Yuneva M (2020). Adapt and conquer: metabolic flexibility in cancer growth, invasion and evasion. Mol Metab.

[33] Paredes F, Williams HC, San Martin A (2021). Metabolic adaptation in hypoxia and cancer. Cancer Lett.

[34] Alvarez-Meythaler JG, Garcia-Mayea Y, Mir C, Kondoh H, LLeonart ME (2020). Autophagy takes center stage as a possible cancer hallmark. Front Oncol.

[35] van Zijl F, Krupitza G, Mikulits W (2011). Initial steps of metastasis: cell invasion and endothelial transmigration. Mutat Res.

[36] Roussos ET, Condeelis JS, Patsialou A (2011). Chemotaxis in cancer. Nat Rev Cancer.

[37] Wang Q, Shao X, Zhang Y (2023). Role of tumor microenvironment in cancer progression and therapeutic strategy. Cancer Med.

[38] Popova NV, Jücker M (2022). The functional role of extracellular matrix proteins in cancer. Cancers (Basel).

[39] Strowitzki MJ, Cummins EP, Taylor CT (2019). Protein hydroxylation by hypoxia-inducible factor (HIF) hydroxylases: unique or ubiquitous?. Cells.

[40] Yu F, White SB, Zhao Q, Lee FS (2001). HIF-1alpha binding to VHL is regulated by stimulus-sensitive proline hydroxylation. Proc Natl Acad Sci U S A.

[41] Masoud GN, Li W (2015). HIF-1α pathway: role, regulation and intervention for cancer therapy. Acta Pharm Sin B.

[42] Tarrado-Castellarnau M, de Atauri P, Cascante M (2016). Oncogenic regulation of tumor metabolic reprogramming. Oncotarget.

[43] Mylonis I, Chachami G, Simos G (2021). Specific inhibition of HIF activity: can peptides lead the way?. Cancers (Basel).

[44] Gavas S, Quazi S, Karpiński TM (2021). Nanoparticles for cancer therapy: current progress and challenges. Nanoscale Res Lett.

[45] Gillespie DL, Aguirre MT, Ravichandran S (2015). RNA interference targeting hypoxia-inducible factor 1α via a novel multifunctional surfactant attenuates glioma growth in an intracranial mouse model. J Neurosurg.

[46] Schönberger T, Fandrey J, Prost-Fingerle K (2021). Ways into understanding HIF inhibition. Cancers (Basel).

[47] Stein CA (2001). The experimental use of antisense oligonucleotides: a guide for the perplexed. J Clin Invest.

[48] Piorecka K, Kurjata J, Stanczyk WA (2022). Acriflavine, an acridine derivative for biomedical application: current state of the art. J Med Chem.

[49] Ricker JL, Chen Z, Yang XP, Pribluda VS, Swartz GM, Van Waes C (2004). 2-methoxyestradiol inhibits hypoxia-inducible factor 1alpha, tumor growth, and angiogenesis and augments paclitaxel efficacy in head and neck squamous cell carcinoma. Clin Cancer Res.

[50] Selvaraju V, Parinandi NL, Adluri RS, Goldman JW, Hussain N, Sanchez JA, Maulik N (2014). Molecular mechanisms of action and therapeutic uses of pharmacological inhibitors of HIF-prolyl 4-hydroxylases for treatment of ischemic diseases. Antioxid Redox Signal.

[51] Zhang YB, Wang X, Meister EA (2014). The effects of CoCl2 on HIF-1α protein under experimental conditions of autoprogressive hypoxia using mouse models. Int J Mol Sci.

[52] Ben Mousa A (2008). Sorafenib in the treatment of advanced hepatocellular carcinoma. Saudi J Gastroenterol.

[53] Hao Z, Sadek I (2016). Sunitinib: the antiangiogenic effects and beyond. Onco Targets Ther.

[54] Zhang J, Chen B, Gan C, Sun H, Zhang J, Feng L (2023). A comprehensive review of small interfering RNAs (siRNAs): mechanism, therapeutic targets, and delivery strategies for cancer therapy. Int J Nanomedicine.

[55] Chen X, Mangala LS, Rodriguez-Aguayo C, Kong X, Lopez-Berestein G, Sood AK (2018). RNA interference-based therapy and its delivery systems. Cancer Metastasis Rev.

[56] Lundstrom K (2020). Viral vectors applied for RNAi-based antiviral therapy. Viruses.

[57] Janji B, Chouaib S (2022). The promise of targeting hypoxia to improve cancer immunotherapy: mirage or reality?. Front Immunol.

[58] Shiravand Y, Khodadadi F, Kashani SM (2022). Immune checkpoint inhibitors in cancer therapy. Curr Oncol.

[59] Sharpe M, Mount N (2015). Genetically modified T cells in cancer therapy: opportunities and challenges. Dis Model Mech.

[60] Conry RM, Westbrook B, McKee S, Norwood TG (2018). Talimogene laherparepvec: first in class oncolytic virotherapy. Hum Vaccin Immunother.

[61] Song CW, Kim H, Cho H (2022). HIF-1α inhibition improves anti-tumor immunity and promotes the efficacy of stereotactic ablative radiotherapy (SABR). Cancers (Basel).

[62] Czajka-Francuz P, Prendes MJ, Mankan A (2023). Mechanisms of immune modulation in the tumor microenvironment and implications for targeted therapy. Front Oncol.

